# Real-World Treatment Sequences and Outcomes Among Patients With Non-Small Cell Lung Cancer (RESOUNDS) in the United States: Study Protocol

**DOI:** 10.2196/resprot.7750

**Published:** 2017-10-11

**Authors:** Lisa M Hess, David M Kern, Gebra Cuyun Carter, Katherine Winfree, Liya Wang, Angelina Sontag, Ana B Oton

**Affiliations:** ^1^ Eli Lilly and Company Indianapolis, IN United States; ^2^ HealthCore, Inc Wilmington, DE United States

**Keywords:** cancer, non-small cell lung cancer, chemotherapy, regimen, line of therapy, treatment sequence, progression-free survival, overall survival, real-world data

## Abstract

**Background:**

Survival outcomes are related to treatment choices in a line of therapy and to treatment sequences across all lines of therapy.

**Objective:**

The Real-World Treatment Sequences and Outcomes among Patients with NSCLC (RESOUNDS) study is designed to (1) evaluate treatment sequences used for patients who receive at least two lines of therapy for non-small cell lung cancer (NSCLC) in the United States and (2) evaluate patient outcomes in terms of progression-free and overall survival related to treatment sequencing. Additional objectives include the evaluation of symptoms, comorbidities, and health care resource utilization and costs.

**Methods:**

Patients will be censored at loss to follow-up due to leaving the health plan or reaching the end of the study period.

**Results:**

This study is ongoing.

**Conclusions:**

The RESOUNDS cohort study is a novel approach to building a comprehensive dataset that mimics a prospective observational study using linked patient-level data from four real-world data sources. This study will provide timely information on the sequencing of treatments for patients with NSCLC.

## Introduction

Lung cancer is the second most common cancer in the United States and the leading cause of cancer-related death, with an estimated 158,040 Americans dying in 2016 from the disease [1]. The two types of lung cancer are small cell and non-small cell lung cancer (NSCLC)—NSCLC accounts for roughly 83% of cases [2]. The US Food and Drug Administration recently approved several novel biologic agents (eg, ramucirumab, nivolumab, and pembrolizumab) [3-6]; as a result, the treatment of NSCLC is rapidly evolving. However, the appropriate timing and most effective sequence of these new agents in the care of patients remains unknown. Previously, care was delivered in distinct lines of therapy, as it was unknown if the patient would be able to continue treatment over time. However, with the advent of newer agents demonstrating improved overall survival outcomes in the postprogression setting, providers now can consider treatment strategies over time. As with many other cancers, lung cancer is increasingly being treated as a chronic condition. As novel agents continue to demonstrate improved survival outcomes in NSCLC, additional data are needed across lines of therapy to identify the optimal sequencing of these agents for the optimization of patient care.

The study of treatment sequences in a randomized controlled trial (RCT) would be extremely challenging, due to not knowing which patients will progress to a second line and to the treatment heterogeneity used in current practice settings across lines of therapy. A sequence can be studied only in patients who receive more than one line of therapy. Recent data suggest that less than half of NSCLC patients will receive a second line of therapy [7]. As a result, a randomized trial would have to double the enrolling patient size beyond the number necessary to ensure statistical power to obtain a sufficient sample of evaluable patients receiving a sequence of agents. Further, for data to be relevant to clinical decision makers, the treatment choices should reflect those available. An RCT of multiple treatment arms would similarly require that sample sizes be increased to ensure that all relevant treatments be applied consistently across the population. Alternatively, prospective observational trials may be conducted that do not rely on randomization but instead permit usual treatment practices to occur as data are being collected. This is appealing to providers and patients who wish to maintain the ability to make treatment choices based on preferences for care. However, a prospective observational trial would still require a very large study population to be enrolled and followed over time, further increasing the time and cost of such a study. The research team has designed the current study as an alternative to these costly and long-term prospective trial designs. The **Re**al-World Treatment **S**equences and **Ou**tcomes among Patients with **NS**CLC **(**RESOUNDS) study was designed to mimic a prospective observational study by using longitudinal data from large pre-existing databases. The enrollment date (termed “index date” for this study) is defined as the start of second-line therapy, and real-world data sources (eg, medical records, claims data) are being culled to create a longitudinal de-identified patient-specific study record retrospectively and contemporaneously from the time of initial diagnosis through the end of the follow-up period.

The primary objective of this study is to describe treatment sequences used for patients who receive at least two lines of therapy for NSCLC in the United States, while secondary objectives include outcomes of survival, disease progression and response, health care resource utilization and costs, and factors associated with treatment decision making. This study integrates multiple pieces of data (ie, medical records, claims, oncology clinical care pathway data, and death index data) in order to gain depth of data related to clinical and economic outcomes as well as to provide a wider breadth of information than is feasible in an RCT or traditional prospective observational trial. This novel approach to mimicking a prospective trial with pre-existing data sources is expected to speed the timing of generating robust outcome data, minimize participant burden, and increase the generalizability of findings to the broader NSCLC population in the United States.

HealthCore, a wholly owned subsidiary of Anthem, Inc., explores health care claims data from Anthem members and investigates health plan clinical cancer care data collected from oncologists participating in a quality improvement initiative designed to provide members diagnosed with cancer access to quality, evidence-based, cost-effective medical care (referred to as “HIRE Oncology”). The quality improvement program provides treating physicians enhanced reimbursement when the treatment regimen is aligned with National Comprehensive Cancer Network and/or American Society of Clinical Oncology guidelines. This program provides physician incentives to use treatment regimens for each line of therapy that are included in the guideline. Additionally, medical records will be reviewed for data not available in other sources (eg, imaging reports, results of biomarker testing). The use of these data has been validated for use in observational research studies [8]. Eli Lilly and Company and HealthCore are conducting this study using these and other linked data resources to better understand the sequencing of care for patients diagnosed with NSCLC.

## Methods

### Study Aims

The overarching goal of the RESOUNDS study is to better understand treatment sequences among patients who received at least two lines of therapy for NSCLC. To achieve this goal, the following specific aims will be pursued for the overall population as well as for specific treatment sequences: (1) describe baseline demographic and clinical characteristics, (2) evaluate clinical outcomes (eg, Eastern Cooperative Oncology Group [ECOG] or Karnofsky performance status, tumor response, disease progression, symptoms, and survival), (3) evaluate health care resource utilization (eg, hospitalizations, emergency room visits, hospice and long-term care, medication use, radiation therapy, imaging) and costs, and (4) evaluate the factors associated with selection of and changes in treatment, including treatment changes and discontinuation of therapy. An exploratory objective will evaluate differences in overall survival among specific subgroups of interest, such as treatment regimen by line of therapy, histology (squamous vs nonsquamous), and treatment sequences.

### Design and Data Sources

This retrospective, observational cohort study uses the integration of HealthCore Integrated Research Environment (HIRE) Oncology data, which are built through multiple sources of data from the Cancer Care Quality Program, HealthCore Integrated Research Database (HIRD) administrative claims data, and National Mortality Registries, supplemented by medical records data. All procedures for this study have been approved by the New England Independent Review Board, which granted a waiver for the research pursuant to 45 CFR (Code of Federal Regulations) §164.512(i) on June 30, 2016.

### HealthCore Integrated Research Environment

**Figure 1:**
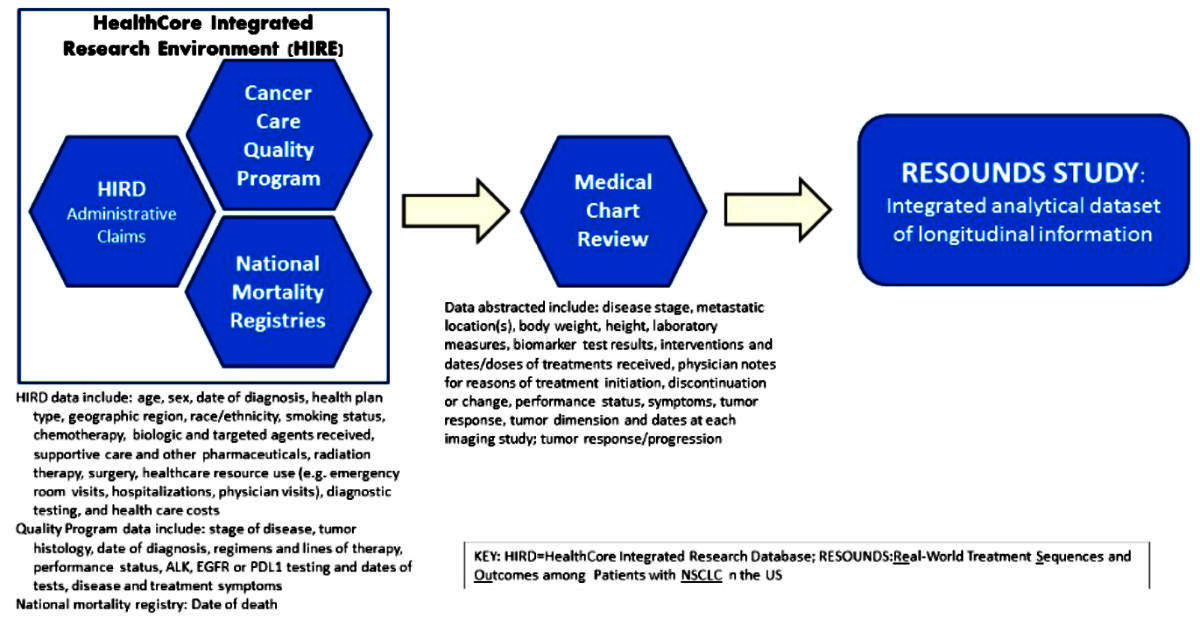
Data sources used for this study.

**Figure 2:**
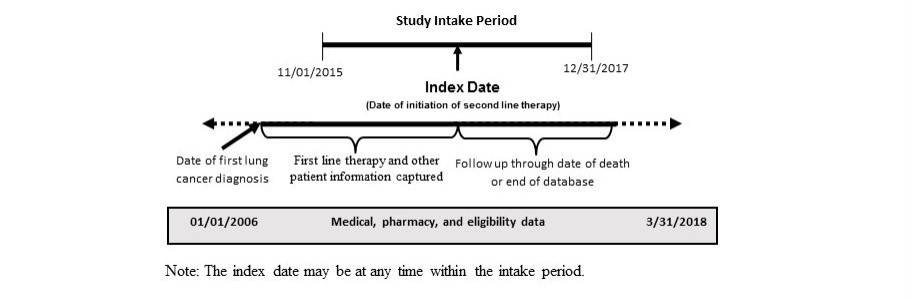
Study time frame.

The HIRE contains a large administrative health care database that can be linked to data sources, including inpatient and outpatient medical records, national vital statistics records, member and provider surveys, and point-of-care clinical data to provide a fully integrated, comprehensive dataset. This study will utilize multiple data sources from within HIRE: (1) claims data from HIRD, (2) Cancer Care Quality Program data, (3) National Mortality Registry data, and (4) data from medical records. Figure 1 illustrates the study design, and Figure 2 demonstrates the data sources proposed for this study.

### HealthCore Integrated Research Database

HIRD is a single payer insurance database that contains administrative health care claims integrated across data sources and types (ie, professional claims, facility claims, outpatient pharmacy claims, outpatient laboratory results, and enrollment information) as well as across years (from 2006 through the most recent month). Data are geographically dispersed and are obtained from 14 Anthem, Inc. affiliated health plans in the Northeastern, Mid-Atlantic, Southeastern, Midwest, Central, and Western regions of the United States, representing members in each of the 50 states. HIRD includes data from January 2006 for all the plans represented in the database. As of December 2015, these data contained information from 38.8 million patient lives with medical and pharmacy eligibility, and 25.8 million patient lives eligible for medical chart review, of which 6.0 million are currently active in the health plan.

#### Cancer Care Quality Program

This program offers evidence-based cancer treatment information enabling physicians to compare planned cancer treatment regimens against evidence-based clinical criteria [8]. The program has identified certain cancer treatment pathways, selected based on current clinical evidence, published literature, and national guideline recommendations, which have shown to be efficacious, less toxic, and cost effective. The physicians participating in the program receive additional reimbursement per patient for prescribed treatment regimens that align with the identified pathway, encouraging evidence-based quality care for the patients and cost benefits for the physicians. Data are obtained when physicians request approval for this pathway-based enhanced reimbursement as well as prior authorization for the various cancer treatments. As of September of 2015, the program has been implemented in all of the Anthem health plans. Cancer treatment pathways collect clinical, demographic, and treatment data for the three highest volume cancers, namely breast, lung, and colorectal. These data are available for a subset of individuals in HIRD via direct patient linkage.

The following clinical information is collected for the Cancer Quality Care Program and is integrated with the medical and pharmacy claims data contained within HIRD: cancer type, cancer stage, tumor biomarkers (eg, epidermal growth factor receptor [EGFR], anaplastic lymphoma kinase [ALK] status), line of treatment (ie, adjuvant/post-operative, first line, second line, third line or more, maintenance), and physician specialty. Since the program was rolled out sequentially in the various Anthem health plans over 15 months, the number of patients has incrementally increased over time. All Anthem health plans are participating as of the start date of this study.

HIRD is hosted on servers contained within HealthCore’s Data Coordination Center, which is located in a locked suite within HealthCore’s facility. Access to this database is through password-protected, 128-bit Secure Socket Layer (SSL) encrypted connections.

#### National Mortality Registry

Data from HIRD are linked to mortality registries via patient identifiers to determine date of death using the Death Master File (DMF) provided by the Social Security Administration (SSA). These data include greater than 80 million individual recorded deaths reported to the SSA (approximately 95% of all deaths that occur in the United States). Cause of death cannot be determined using the DMF. Data from the SSA will also be supplemented with data from HIRD that contain discharge data indicating death for those patients who died in the hospital setting.

#### Medical Record Data

Patients will be identified from HIRD claims data, and their medical records will be targeted for abstraction by contacting the patients’ providers. A chart abstraction form will provide the chart abstractor with information on what data are to be abstracted from the charts and forwarded to the study team. Charts are successfully abstracted for approximately 65% of patients on average. Thus, not all patients will have available chart data.

To help ensure the consistency of data collection, the chart reviewers (eg, nurses, pharmacists, and physicians) have received detailed training on the study’s design. Training for abstraction also includes a detailed review of the standardized data collection forms approved by the New England Institutional Review Board. Clinical information that is abstracted or redacted from the medical and hospital charts is entered into a secure electronic database with a masked identifier so that it can be matched with corresponding electronic claims data without the use of individually identifiable information.

### Eligibility Criteria

The eligible patient population must meet the following criteria:

- Patients with squamous or nonsquamous metastatic NSCLC who have initiated second-line therapy with pemetrexed, ramucirumab, docetaxel, pembrolizumab, nivolumab, gemcitabine, paclitaxel, necitumumab, vinorelbine, cisplatin, carboplatin, bevacizumab, afatinib, nab-paclitaxel, atezolizumab, and/or erlotinib.
- Patients must have ≥1 medical claim with a diagnosis for lung cancer (*International Statistical Classification of Diseases*, 9^th^ or 10^th^ Revision, Clinical Modification [ICD-9-CM] codes 162.2x-162.9x or ICD-10-CM codes C34.xx) prior to initiation of second-line therapy.
- Second-line therapy must be initiated between November 1, 2015, and December 31, 2017.
- For claims data, there must exist at least 90 days of health plan enrollment prior to the date of initial first-line chemotherapy to ensure a complete patient record is available.
- Patients age ≥18 at the initial diagnosis of lung cancer.

Patients with small-cell lung cancer and patients <18 years old as of the earliest observed lung cancer diagnosis date are excluded.

After determining eligibility, patient medical records will be reviewed to ensure that the patient has NSCLC due to the nonspecific nature of ICD-CM-9 and ICD-CM-10 codes alone and that the treatment in the medical record verifies what is identified in the claims data to ensure the patient has received at least two lines of therapy. This ensures an advanced/metastatic cohort for this study. Patients will be identified from the HIRD and HIRE Oncology datasets, with verification from the medical record.

### Study Timeframe

Patients will be identified based on initiation of second-line therapy between November 1, 2015, and December 31, 2017 (referred to as the “intake period”). The “index date” for each patient (similar to the enrollment date in a prospective observational trial) is defined as the start date of the second line of therapy (line of therapy is defined by the treating oncologist in the HIRE Oncology dataset). Patient history will be examined as far back as the first observed diagnosis for lung cancer (identified via claims or medical records, where applicable). Patients will be followed forward in time for as long as possible (eg, until death or disenrollment from their health plan or end of the study period). The final claims data base will be created in June 2018, at which point data will be available through at least March 2018. The RESOUNDS study design is shown in Figure 1.

### Variables and Timing

Due to the observational nature of this study, data will be collected from a variety of sources as the events occurred, rather than at prespecified time points. All instances of data consistent with the variables of interest will be included with their respective dates from the patient record. No restrictions or limitations are made on the number or frequency of variables collected. The data to be captured include patient demographic and clinical characteristics (eg, age, sex, height, stage of disease, tumor histology, date of diagnosis, health plan type, geographic region, race/ethnicity, smoking status), body weights and laboratory measures, chemotherapy, biologic and targeted agents received (including doses and dates received), regimens and lines of therapy, supportive care and other pharmaceuticals received, radiation therapy and dates received, surgery and procedures and dates, physician notes for reasons of treatment initiation, discontinuation or change, performance status, symptoms, tumor response as physician assessed, tumor response per Response Evaluation Criteria in Solid Tumors (RECIST) criteria [9]; largest tumor dimension at each imaging study; date of death; ALK, EGFR, or programmed death-ligand 1 (PD-L1) testing and dates of tests; disease and treatment symptoms; health care resource use (eg, emergency room visits, hospitalizations, physician visits); diagnostic testing; and health care costs (Figure 1). Each of the variables in this study is collected from the previously described data sources used within the study to ensure accuracy. For example, claims data (eg, HIRD dataset) do not contain clinical data such as disease stage or histology. Therefore, these data are linked to the HIRE dataset (which contains both stage and histology as mandatory collection fields) and are further verified in the patient medical record. Similarly, chemotherapy medications are identified in claims data using generic product identifier and Healthcare Common Procedure Coding System values. First-line therapies are further verified in the medical record, and subsequent therapies are verified in the HIRE data. For other variables, such as resource utilization (eg, hospitalization, emergency room visits) and costs, the HIRD data (eg, claims) are the primary source, as this dataset records all health care‒related activity submitted to the insurer from any provider for care received by the patient at any health care setting.

### Statistical Analysis Plan

Calculations were performed to determine the statistical power to detect overall survival differences over varying sample sizes. Two scenarios were modeled: (1) the median survival is 6 months in group A and 9 months in group B, and (2) median survival times are 6 months in group A and 12 months in group B. Other assumptions include 12 months of accrual and 12 months of follow-up. Table 1 presents the sample sizes from 10-300 with their resulting power assuming equal number of patients in each treatment group, significance level at .05 in a two-sided test, and assuming no loss to follow-up. It is expected that approximately 400 patients will be included in this study, and the size of the subgroups (eg, treatment regimens and sequences) for comparison will be a result of the treatment patterns and practices of their treating oncologists. Assignment of treatment is not being made in this observational study. The survival analyses are exploratory in nature in this study, and the magnitude of results, rather than statistical significance, will be used to inform potential future studies examining survival in more detail with larger and sufficient sample sizes.

Descriptive analysis will be performed to describe treatment sequences among NSCLC patients with at least two lines of therapy. Univariate statistics will include means, standard deviations, medians, and interquartile range for continuous variables, and relative frequencies and percentages for categorical variables. Kaplan-Meyer survival analysis will be used to estimate the time to event metrics for the entire study population and by each second-line regimen cohort. Additional subgroups include histology (squamous vs nonsquamous), line of therapy, and treatment sequences (eg, sequencing of immunotherapy, chemotherapy, biologic/targeted therapy drug classes). While the use of multiple data bases is expected to minimize the rate of missing data, there is the risk of missing data. For these variables, the number and percent missing will be reported, but no imputation will be made for those variables that cannot be confirmed in the medical record.

The decision to move from one treatment regimen to the next will be analyzed using classification and regression tree analysis. The goal will be to use this recursive partitioning technique to find the factors most highly associated with treatment changes. This will be done for treatment changes overall (without differentiating treatment lines) and by each treatment line (eg, the first treatment change will be the start of second line; the second treatment change will be the start of third-line therapy). Furthermore, within each treatment change the outcome will be the specific change in therapy from line n to line n+1, where n=1, 2, 3. Analysis of specific treatment sequences from one line to the next will be limited to sequences with sufficient sample size for analysis.

Statistical testing will be performed for the outcome of overall survival for select comparison groups. The survival analyses are exploratory in nature and statistical testing will be performed only if there are at least 50 patients in each group. Survival analyses will adjust for covariates. Due to the limited number of patients expected in some of the subgroups, only 5-10 covariates will be included in the models to avoid overfitting the data. The following demographic covariates will be included in all statistical models: patient age, sex, health plan type, geographic region, and Deyo Comorbidity Index score [10]. Additional potential covariates to be included in each model will be selected according to their bivariate statistical significance (*P*<.05) with the outcome. These potential covariates include disease stage at initial diagnosis, smoking status, race/ethnicity, body mass index, and date of first diagnosis. No adjustment will be made for multiple testing.

Interim analyses for the descriptive/noncomparative endpoints are planned at semi-annual intervals through 2018. These descriptive data may be useful to inform the development of new trials or may be informative as to the changing nature of NSCLC care during the study period.

**Table 1:** Calculated statistical power for varying sample sizes for two different survival scenarios, assuming no loss to follow-up.

Median survival time^a^	Total N	Power
9	10	.089
9	20	.128
9	30	.169
9	40	.210
9	50	.250
9	60	.291
9	80	.369
9	100	.443
9	120	.512
9	140	.576
9	160	.633
9	180	.684
9	200	.729
9	220	.769
9	240	.804
9	260	.834
9	280	.860
9	300	.883
9	320	.902
9	350	.925
9	370	.938
9	400	.953
12	10	.158
12	20	.269
12	30	.376
12	40	.475
12	50	.564
12	60	.642
12	80	.765
12	100	.851
12	120	.908
12	140	.944
12	160	.967
12	180	.981
12	200	.989
12	220	.994
12	240	.996
12	260	.998
12	280	.999
12	300	>.999
12	320	>.999
12	350	>.999
12	370	>.999
12	400	>.999

^a^Compared to a group of patients with median survival of 6 months using log-rank test for equality of survival curves. Power was calculated assuming equal number of patients in each treatment group, significance level at .05 in a two-sided test, and no loss to follow-up.

## Discussion

### Principal Considerations

Observational research can provide highly generalizable data and can be used to conduct comparative effectiveness research as a complement to RCTs [11]. When designed appropriately, data from retrospective sources can be used to address scientific questions in a timely and cost-effective manner. The RESOUNDS study has been designed to address complex questions in the care of patients with NSCLC during a time where treatment strategies are changing and evolving. Rather than predefine the sequences of interest, this study will examine how physicians are treating patients and the outcomes associated with these sequences. As with observational study designs, these data will result in hypothesis-generating rather than definitive results from a priori hypotheses. However, these data may be used to guide the development of future RCTs, to better understand the optimal sequences, and to inform evidence-based medicine. At this time, little is known about the sequence of care for NSCLC, and this study will provide a robust dataset from which to address these and future research questions.

### Limitations

Limitations of these data may include undercollected or missing data. For example, ECOG performance status, while a standard data item in cancer RCTs, is not routinely collected in patient care settings and may not be available for all patients. Similarly, tumor growth and progression is not commonly defined per RECIST criteria outside of a clinical trial setting and may not be applied to all imaging reports. Therefore, some of the research questions of interest may have underpopulated data. Similarly, while PD-L1 status may be an important research question, it is not currently being evaluated in the majority of NSCLC patients. This may result in a relatively small population that cannot be statistically compared or may result in the inability to evaluate patient outcomes by PD-L1 status. Therefore, as the data are not known until the time of analysis, no adjustments will be made to any study objective and no imputations will be made for missingness. As the comparative analyses are largely exploratory in nature, these will be conducted only if there is a sufficient sample with complete data.

### Conclusion

The RESOUNDS cohort study is a novel approach to building a comprehensive dataset that mimics a prospective observational study using real-world data sources. This study will provide timely information on the sequencing of treatments for patients with NSCLC.

## Abbreviations

ALK: anaplastic lymphoma kinase
DMF: Death Master File
ECOG: Eastern Cooperative Oncology Group
EGFR: epidermal growth factor receptor
HIRD: HealthCore Integrated Research Database
HIRE: HealthCore Integrated Research Environment
ICD-9-CM, ICD-10-CM: International Statistical Classification of Diseases, 9th or 10th Revision, Clinical Modification
NSCLC: non-small cell lung cancer
PD-L1: programmed death-ligand 1
RCT: randomized controlled trial
RECIST: Response Evaluation Criteria in Solid Tumors
RESOUNDS: Real-World Treatment Sequences and Outcomes among Patients with NSCLC study
SSA: Social Security Administration
